# Comparative mRNA-Seq Analysis Reveals the Improved EPS Production Machinery in *Streptococcus thermophilus* ASCC 1275 During Optimized Milk Fermentation

**DOI:** 10.3389/fmicb.2018.00445

**Published:** 2018-03-13

**Authors:** Qinglong Wu, Nagendra P. Shah

**Affiliations:** Food and Nutritional Science, School of Biological Sciences, The University of Hong Kong, Pokfulam, Hong Kong

**Keywords:** exopolysaccharide, *Streptococcus thermophilus*, milk fermentation, transcriptome, pH

## Abstract

Exo-polysaccharide (EPS) produced by dairy starters plays critical roles in improving texture and functionalities of fermented dairy products. One of such high EPS producers, *Streptococcus thermophilus* ASCC 1275 (ST1275) was used as a model dairy strain to understand the stimulation of its EPS production under optimal milk fermentation conditions. The mRNA-seq analysis and targeted pathway analysis indicate that genes associated with lactose (milk sugar) catabolism, EPS assembly, proteolytic activity, and arginine/methionine/cysteine synthesis and transport in ST1275 were significantly up-regulated under the optimized conditions of pH 5.5, 40°C, or WPI supplementation compared to that of pH 6.5 and 37°C, respectively. This indicates that genes involved in above metabolisms cooperate together for improving EPS yield from ST1275. This study provides a global view map on potential targeted pathways and specific genes accounted for enhanced EPS production in *Str. thermophilus* and that could be modulated by fermentation conditions.

## Introduction

Food-grade exo-polysaccharide (EPS) from lactic acid bacteria (LAB) and bifidobacteria plays important roles in improving the texture and functionalities of foods (Welman and Maddox, 2003; Galle and Arendt, 2014). There are numerous reports on the isolation and identification of EPS producers, optimization of EPS production and chemical and functional characterization of EPS (Ruas-Madiedo and De Los Reyes-Gavilán, 2005; Tabibloghmany and Ehsandoost, 2014). Since past decades, EPS production from dairy starter bacteria has drawn great attentions from dairy industry due to its important value-added functions such as texture-modifying property, immunomodulation and novel prebiotic for gut microbiota (Badel et al., 2011; Tabibloghmany and Ehsandoost, 2014; Salazar et al., 2016). However, there are challenges to optimize dairy fermentation process for EPS production due to limitations for manufacturing fermented dairy products such as cheese and yogurt. Thus, seeking for high EPS-producing dairy starter bacteria, i.e., *Streptococcus thermophilus* (Iyer et al., 2010), has become a promising strategy to make EPS-rich fermented milks.

Several studies have demonstrated high EPS production from non-starter lactic acid bacteria (NSLAB) such as *Lactobacillus casei* group, *Lb. acidophilus, Lb. helveticus, Lb. brevis*, and *Lb. plantarum* (Welman and Maddox, 2003; Tabibloghmany and Ehsandoost, 2014). For example, *Lb. rhamnosus* RW-9595M produced the highest amount of EPS in chemically defined medium among the reported strains of LAB and bifidobacteria (Bergmaier et al., 2004). Although NSLAB strains have been reported to improve the quality of some fermented dairy foods (Leroy and De Vuyst, 2004; Settanni and Moschetti, 2010), these NSLAB strains may only be adopted as adjunct starters due to their weak proteolytic activities and low acidifying rates (Buckenhüskes, 1993; Sasaki et al., 1995). Thus, numerous strains of typical dairy starters including *Str. thermophilus, Lb. delbrueckii* ssp., *bulgaricus* (*Lb. bulgaricus*), and *Lactococcus lactis* ssp. *lactis* (*Lc. lactis*) have been characterized for EPS production but their yields were not high (Frengova et al., 2000). Among them, *Str. thermophilus* ASCC 1275 (ST1275), a conventional dairy starter, has been identified in our pioneer study documented in 2003 as a high EPS producer in milk, and its EPS production could be simply improved by adjusting the fermentation conditions such as pH, temperature or supplementing milk with limited amount of whey protein concentrate/isolate (WPC or WPI), a by-product from cheese-making process (Zisu and Shah, 2003). Characteristics of EPS from ST1275 have been extensively studied in our lab for use in fermented milk products (Amatayakul et al., 2006a,b; Purwandari et al., 2007; Li and Shah, 2014, 2016).

Our attention was drawn when slight changes in the cell viability of ST1275 cultivated in milk under different fermentation conditions exhibited a major shift in EPS production (Figure S1, Supplementary Material) by this organism (Zisu and Shah, 2003). This motivated us to understand the intracellular changes in regards to EPS biosynthesis in ST1275. Recently, our genomic study provided detailed insights into gene features that are associated with nucleotide sugar synthesis and EPS assembly in ST1275, It is also interesting that ST1275 could produce the highest amount (refer to Supplementary Table 6 of the reference) of EPS in milk among the reported strains of *Str. thermophilus* (Wu et al., 2014). Due to the importance of *Str. thermophilus* in dairy industry (Iyer et al., 2010), ST1275 was used as a model strain of high EPS producer in this study to understand the role of environmental conditions in shaping bacterial physiology and EPS biosynthetic machinery. The mRNA-seq analysis was applied to profile gene expression patterns of ST1275 cultivated in milk under different fermentation conditions that have been documented in our previous study (Zisu and Shah, 2003).

## Materials and methods

### Bacterial cultivation and batch milk fermentation

*Streptococcus thermophilus* ASCC 1275 (ST1275) was cultivated in Difco™ M17 broth (BD company, Franklin Lakes, NJ, USA) containing 1% (w/v) lactose at 37°C for 18 h prior to the inoculation (1%; v/v) into 10% (w/v) reconstituted skim milk (RSM). Milk batch fermentations under different pH, temperature and whey protein isolate (WPI) were followed as in our 2003 pioneer study for this strain (Zisu and Shah, 2003). The RSM medium was autoclaved at 120°C for 15 min. Milk fermentation was carried out in an assembled bioreactor—GLS 80® stirred reactor (DURAN Group, Mainz, Germany). The pH probe was sterilized by 1 N NaOH solution and was then inserted into the bioreactor through one of the ports in screw cap. Another two ports were connected to 6 M NaOH and 50% (wt/vol) citric acid solutions which were used for adjusting the pH of milk. Four conditions were selected for milk fermentation based on our previous study: condition 1 (Cd1)—pH 6.5 and 37°C; condition 2 (Cd2)—pH 5.5 and 37°C; condition 3 (Cd3)—pH 5.5 and 40°C; condition 4 (Cd4)—pH 5.5 and 37°C with 0.5% (wt/vol) WPI supplementation to the RSM. The EPS production from ST1275 from above conditions was observed after 24 h: Cd1−255 mg/L; Cd2−458 mg/L; Cd3−622 mg/L; Cd4−1,029 mg/L (Zisu and Shah, 2003). The milk fermentation was stopped after 6 h since the cells were in late log-growth phase. One volume of fermented milk after sampling was immediately treated with 0.33 volume of 1 M trisodium citrate (Derzelle et al., 2005); bacterial cell pellet were harvested by centrifugation and stored at −80°C for further RNA extraction. Three independent fermentation experiments were performed for each condition.

### Total RNA extraction and mRNA-Seq procedure

Total RNA from bacterial cell pellet was extracted with Ambion's RiboPure-Yeast kit as per manufacturer's instructions. Extra DNase I treatment was necessary to remove any genomic DNA. Then, RNA integrity number (RIN) was obtained from Agilent 2100 Bioanalyzer, all extracted RNA samples have RIN score above nine indicating low RNA degradation; these samples were qualified for RNA-seq and were sent to BGI Tech Solutions (Hong Kong) Co., Ltd. for downstream sequencing process. Basically, ribosomal RNA (rRNA) was removed before library constructions followed by mRNA fragmentation, cDNA synthesis and fragment enrichment; then libraries were loaded into the Illumina HiSeq4000 platform for sequencing. The transcriptome datasets of the current study are available in the Sequence Read Archive (SRA) database in National Center for Biotechnology Information under accession numbers from SRX3029248 to SRX3029259.

### Bioinformatic and statistical analyses

The filtering process of raw sequencing reads was carried out by BGI Tech Solutions (Hong Kong) Co., Ltd. resulting in about 2 gigabases of clean Illumina reads per sample. The quality threshold was set to 20 for filtering by BGI. Since *Str. thermophilus* ST1275 has been fully sequenced; read alignment/mapping was achieved by the package Tophat version 2.1.1 with very sensitive mode (read mismatches: 2) with the usage of Bowtie version 2.1.0 (Kim et al., 2013). The read counts per gene per sample were generated with HTSeq package (version 0.9.1) after the sorting procedure for accepted hits in BAM format with samtools (version 1.3.1) (Anders et al., 2015). The edgeR's quasi-likelihood pipeline (Robinson et al., 2010) took read count table for downstream analysis and the key parameters used are as follows: genes were excluded for analysis if it was not expressed at a count-per-million (CPM) above 0.5 in at least two libraries; trimmed mean of M values (TMM) normalization approach was performed to eliminate composition biases among all libraries (Robinson and Oshlack, 2010); log_2_CPM value was calculated for generating heatmaps, multi-dimensional scaling (MDS) plot, principal component analysis (PCA) plot, and downstream statistical analysis; trended dispersion was estimated for quasi-likelihood F-tests. False discovery rate (FDR) has been introduced to improve the statistical analysis for differentially expressed genes (DEGs). Gene with a fold-change significantly above 1.0 at a FDR cut-off of 5% is considered as a DEG.

### Reverse-transcriptase quantitative PCR assay

In order to validate gene expression level generated from RNA-seq, 20 genes (Cd2 vs. Cd1, Cd3 vs. Cd2, and Cd4 vs. Cd2) were selected for qPCR validation; these genes should have varying levels (log_2_ fold-change) of gene expression; we also included several key genes involved in EPS biosynthesis and proteolysis in this validation. Total RNA samples were treated with DNase I (Invitrogen) to remove remaining DNA; cDNA synthesis was carried out with High-Capacity RNA-to-cDNA™ Kit (Applied Biosystems). RT-qPCR assays were carried out using SYBR™ Green PCR Master Mix as previously described (Wu et al., 2015). The primers for 20 target genes and reference gene (*tuf*) are listed in Table 1. The amplification efficacy for each pair of primers was in the range of 90–110%, and non-specific amplification products were not detected by both melt curve analysis and agarose gel electrophoresis. Comparative critical threshold method (2^−ΔΔ*Ct*^) was used to calculate relative expression of each targeted gene: Cd1 was used as the reference for Cd2 vs. Cd1 comparison, and Cd2 was used as the reference for Cd3 vs. Cd2 and Cd4 vs. Cd2 comparisons. Each cDNA sample was performed in duplicates (technical repeats) in this assay; total RNA samples isolated from triplicate samples (biological repeats) per one condition were included in qPCR assay; qPCR assay was carried out for the same RNA samples that were also used for RNA-sequencing. The mean values of log2 fold-change (RNA-seq analysis) and -ΔΔCt (qPCR assay) of the selected genes were used for correlation analysis.

**Table 1:** Reverse-transcriptase qPCR primers used in this study.

**Comparison group**	**Gene ID**	**Gene annotation**	**Amplicon size (bp)**	**Forward primer ID**	**Forward primer (5′ → 3′)**	**Reverse primer ID**	**Reverse primer (5′ → 3′)**
Reference gene	T303_03540	tuf	132	3450-F	GTGTCCTTCTTCGTGGTATC	3450-R	GACGTCCACCTTCTTCTTTAG
Cd2 vs. Cd1	T303_00020	Argininosuccinate lyase	117	20-F	CCAGCTTACCAGCCTTATATTC	20-R	GCTCTATTGCCCATGTAACC
	T303_02895	Cystathionine gamma-synthase	94	2895-F	GGTAAGGGTGGGATGATTTC	2895-R	GACTCTCCGCAAAGGTAAAG
	T303_06345	epsM	128	6345-F	GTACGATCCAAAGTCACGTATAG	6345-R	TTACCAGCGAGACCTTACA
	T303_06350	epsL	123	6350-F	TGGCACGAATTTAGGAGTAATAG	6350-R	GCCTTTGCACATAGAGAGTATAG
	T303_08300	Amino acid transport system substrate-binding protein	119	8300-F	GACTCCTTGGTCTCTTTGATG	8300-R	ACCCTTGCCAGCTTTAAC
	T303_01040	Tryptophanyl-tRNA synthetase	138	1040-F	CTGGATCTTCCACACGAATATG	1040-R	TCCCAGGTCTAGATGGTAATG
	T303_01340	Amidophosphoribosyltransferase	121	1340-F	GATTCGTCGTAGCCATAACC	1340-R	TGGATCCAAAGCTGCAATAA
	T303_01945	CTP synthase	121	1945-F	GCTGGACAAGGAATCAAGAA	1945-R	TACCCTGAGCCTGCATATTA
	T303_07280	Citrate synthase	118	7280-F	ACACTCATCGGGTGTAAATG	7280-R	CAACGCAAATGGAACTCAAG
	T303_09710	Glutamine synthetase	117	9710-F	GGAAGTAGTATGCGTCTTGAG	9710-R	GTATTGCCGGTAGTGGTATG
Cd3 vs. Cd2	T303_03150	Fructose operon transcriptional repressor	97	3150-F	CATGCTGTTAAGCTGGTAGA	3150-R	CCAGAGCTGTACTTCCAATAG
	T303_05205	Cell envelope serine proteinase	94	5205-F	AACCACAGTCAGGCAATAAG	5205-R	CTGTCTGTCCCATTCCATTC
	T303_05515	Glucosamine–fructose-6-phosphate aminotransferase	111	5515-F	CCAGTTGAGCTCGGTATTTC	5515-R	CTGACGACTATCTGCTGTTTC
	T303_07870	Lactose permease	100	7870-F	TGGCGAGTAGGAAGAATTTG	7870-R	CTTCAGGTAGCATGGGTAAAG
	T303_09015	Universal stress protein UspA	95	9015-F	GTGCCACTGGTCTCAATAC	9015-R	ACGAACAACGAGCAAGTC
Cd4 vs. Cd2	T303_02960	Cysteine synthase A	105	2960-F	CAGCTGGATTTGAAGGGTTAG	2960-R	CTGGTAGCGAAGGAATGAAAG
	T303_03905	Peptidase M20	101	3905-F	CCTTCGTTCAGGTGCTATTT	3905-R	CCGTATCCACACCCAAATC
	T303_04075	Homocysteine S-methyltransferase	123	4075-F	GCTGTAGGCATCAACTGTAG	4075-R	CTGACTGTCGCCATCATAAA
	T303_05970	O-acetylhomoserine (thiol)-lyase	135	5970-F	TGAGATTGCTCACAGTCATAAG	5970-R	GTTCCATGTCCACCGATAAA
	T303_01480	Arsenate reductase	113	1480-F	GGTTGAACAAGCATCAACTTC	1480-R	GATGCTCTCAACACCATTCT

## Results

### RNA-seq data production and read mapping/alignment

The results of sequencing data production and read mapping/alignment are shown in Tables 2, 3. As shown in Table 2, about 99% of raw forward read and 97.5% raw reverse read per sample passed the filtering at quality threshold of 20; this resulted in about 10,000,000 pairs of reads totaling 2 gigabases per sample. Among these filtered pairs, about 93.5% of them termed as concordant aligned pairs that were uniquely mapped to the genome of ST1275 (Table 3); this provides enough sequencing depth for identifying DEGs.

**Table 2:** Sequence data production from RNA-seq.

**Condition**	**Sample ID**	**Clean reads**	**Clean bases (bp)**	**Read length (bp; R1&R2)**	**Q20 (%)**	
					**Read 1**	**Read 2**
pH 6.5 and 37°C	Cd1_rep1	20,236,810	2,023,681,000	100	98.54	97.86
	Cd1_rep2	20,268,110	2,026,811,000	100	99.14	97.83
	Cd1_rep3	20,167,946	2,016,794,600	100	99.11	97.89
pH 5.5 and 37°C	Cd2_rep1	22,527,592	2,252,759,200	100	99.06	97.29
	Cd2_rep2	22,539,134	2,253,913,400	100	99.02	97.12
	Cd2_rep3	20,228,350	2,022,835,000	100	99.12	97.84
pH 5.5 and 40°C	Cd3_rep1	20,113,284	2,011,328,400	100	99.13	98.04
	Cd3_rep2	20,255,118	2,025,511,800	100	99.11	97.82
	Cd3_rep3	20,185,884	2,018,588,400	100	99.09	97.61
pH 5.5 and 37°C with WPI	Cd4_rep1	22,502,782	2,250,278,200	100	99.02	96.82
	Cd4_rep2	20,240,064	2,024,006,400	100	99.13	97.92
	Cd4_rep3	22,534,968	2,253,496,800	100	98.99	97.14

**Table 3:** Sequence read mapping/alignment by Bowtie/TopHat.

**Condition**	**Sample ID**	**Clean reads**	**Clean reads (pairs)**		**Mapped**		**Concordant aligned pairs(%)**
			**Read 1**	**Read 2**	**Read 1(%)**	**Read 2(%)**	
pH 6.5 and 37°C	Cd1_rep1	20,236,810	10,118,405	10,118,405	97.10	95.50	94.70
	Cd1_rep2	20,268,110	10,134,055	10,134,055	98.00	95.50	94.90
	Cd1_rep3	20,167,946	10,083,973	10,083,973	96.70	94.20	93.50
pH 5.5 and 37°C	Cd2_rep1	22,527,592	11,263,796	11,263,796	97.30	94.00	93.20
	Cd2_rep2	22,539,134	11,269,567	11,269,567	97.20	93.50	92.70
	Cd2_rep3	20,228,350	10,114,175	10,114,175	97.40	95.10	94.40
pH 5.5 and 40°C	Cd3_rep1	20,113,284	10,056,642	10,056,642	96.30	94.10	93.40
	Cd3_rep2	20,255,118	10,127,559	10,127,559	97.60	95.10	94.50
	Cd3_rep3	20,185,884	10,092,942	10,092,942	97.50	94.60	94.00
pH 5.5 and 37°C with WPI	Cd4_rep1	22,502,782	11,251,391	11,251,391	97.10	92.80	92.00
	Cd4_rep2	20,240,064	10,120,032	10,120,032	97.60	95.20	94.50
	Cd4_rep3	22,534,968	11,267,484	11,267,484	97.20	93.60	92.70

### RNA-seq analysis and RT-qPCR validation

**Figure 1:**
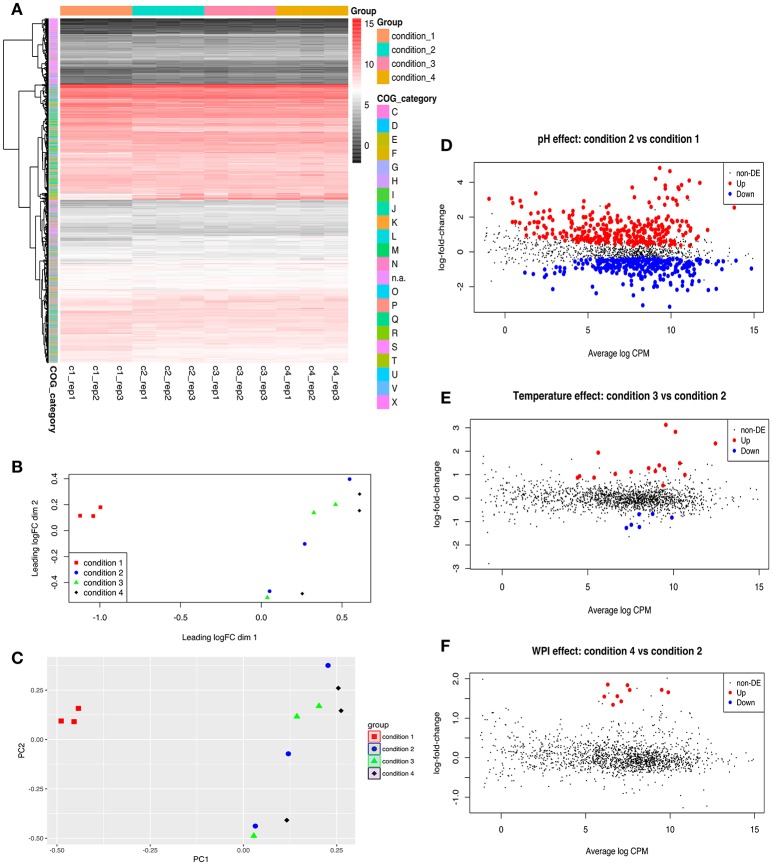
Genome-wide comparative transcriptome analysis of ST1275 under different pH, temperature and milk base**. (A)** Heatmap of gene expression level for samples collected from 4 conditions; genes are retained if they are expressed at a count-per-million (CPM) above 0.5 in at least two samples; expression values are normalized log_2_CPM by using edgeR package. **(B)** visualization of expression profiles (log_2_CPM) of different samples by multi-dimensional scaling (MDS) plot in edgeR package. **(C)** visualization of expression profiles (log_2_CPM) of different samples by principal component analysis (PCA) plot in ggfortify package. **(D)** Detection of DEGs for samples from condition 2 compared to samples from condition 1. **(E)** Detection of DEGs for samples from condition 3 compared to samples from condition 2. **(F)** Detection of DEGs for samples from condition 4 compared to samples from condition 2. Genes with a fold-change significantly above 1.0 at an FDR cut-off of 5% is considered as DEGs. COG denotation: C, energy production and conversion; D, cell cycle control, cell division, chromosome partitioning; E, amino acid transport and metabolism; F, nucleotide transport and metabolism; G, carbohydrate transport and metabolism; H, coenzyme transport and metabolism; I, lipid transport and metabolism; J, translation, ribosomal structure and biogenesis; K, transcription; L, replication, recombination and repair; M, cell wall/membrane/envelope biogenesis; N, cell motility; n.a., not assigned; O, post-translational modification, protein turnover, chaperones; P, inorganic ion transport and metabolism; Q, secondary metabolites biosynthesis, transport and catabolism; R, general function prediction only; S, function unknown; T, signal transduction mechanisms; U, intracellular trafficking, secretion, and vesicular transport; V, defense mechanisms; X, mobilome: prophages, transposons.

General presentations of RNA-seq analysis results are shown in Figure 1, Table 4 and the detailed expression data (log_2_CPM and log-fold-change) is presented in Table S1. Both MDS and PCA plots showed a clear separation of condition 1 from other three conditions suggesting the optimal pH for enhanced EPS production had very significant effect on the transcriptome of ST1275 (Figures 1A–C). The result shows that 292 genes were down-regulated and 308 up-regulated DEGs (Cd2 vs. Cd1) in total, whereas only 23 DEGs (Cd3 vs. Cd2) and 9 DEGs (Cd4 vs. Cd2) were observed in the evaluation of effects of temperature and WPI, respectively (Figures 1D–F). General presentation of DEGs with COG classification is shown in Table 4; the pH decrease (Cd2 vs. Cd1) had dramatically affected on the gene expression profiles with similar numbers of up and down DEGs. It is clear that temperature increase (Cd3 vs. Cd2) and WPI supplementation (Cd4 vs. Cd2) significantly improved the gene expression level of genes classified into carbohydrate (lactose) transport and metabolism and amino acids (arginine, cysteine, and methionine) transport and metabolism, respectively (Table 4). As shown in Table S1, about 1711 mRNA transcripts were detected by RNA-seq, whereas the amount of coding sequences (CDS) of ST1275 were 1694, suggesting the highly sensitive detection of gene transcripts by RNA-seq approach offering genome-scale profiling map.

**Table 4:** Summary of up and down DEGs with COG classification among three comparisons.

**COG category**	**COG description**	**Total genes**	**Cd2 vs. Cd1**		**Cd3 vs. Cd2**		**Cd4 vs. Cd2**	
			**up DEGs**	**down DEGs**	**up DEGs**	**down DEGs**	**up DEGs**	**down DEGs**
C	Energy production and conversion	35	2	17	1	0	0	0
D	Cell cycle control, cell division, chromosome partitioning	21	3	6	0	0	0	0
E	Amino acid transport and metabolism	161	42	33	1	0	6	0
F	Nucleotide transport and metabolism	65	13	21	2	0	0	0
G	Carbohydrate transport and metabolism	47	2	5	6	0	0	0
H	Coenzyme transport and metabolism	49	6	8	0	0	0	0
I	Lipid transport and metabolism	38	3	18	0	0	0	0
J	Translation, ribosomal structure and biogenesis	176	25	31	0	0	0	0
K	Transcription	59	9	9	1	0	0	0
L	Replication, recombination and repair	74	14	13	0	0	0	0
M	Cell wall/membrane/envelope biogenesis	74	10	12	0	0	0	0
N	Cell motility	4	1	2	0	0	0	0
n.a.	COG not assigned	570	102	54	2	4	1	0
O	Post-translational modification, protein turnover, chaperones	50	8	6	0	0	1	0
P	Inorganic ion transport and metabolism	61	13	7	0	0	1	0
Q	Secondary metabolites biosynthesis, transport and catabolism	6	1	0	0	0	0	0
R	General function prediction only	75	12	17	2	1	0	0
S	Function unknown	74	17	10	0	1	0	0
T	Signal transduction mechanisms	37	4	6	0	0	0	0
U	Intracellular trafficking, secretion, and vesicular transport	14	2	4	0	0	0	0
V	Defense mechanisms	45	9	11	2	0	0	0
X	Mobilome-prophages, transposons	36	10	2	0	0	0	0
Total	1,771	308	292	17	6	9	0	

*COG annotation (version COG2014 update) for each gene of ST1275 was downloaded from Integrated Microbial Genomes and Microbiomes*.

**Figure 2:**
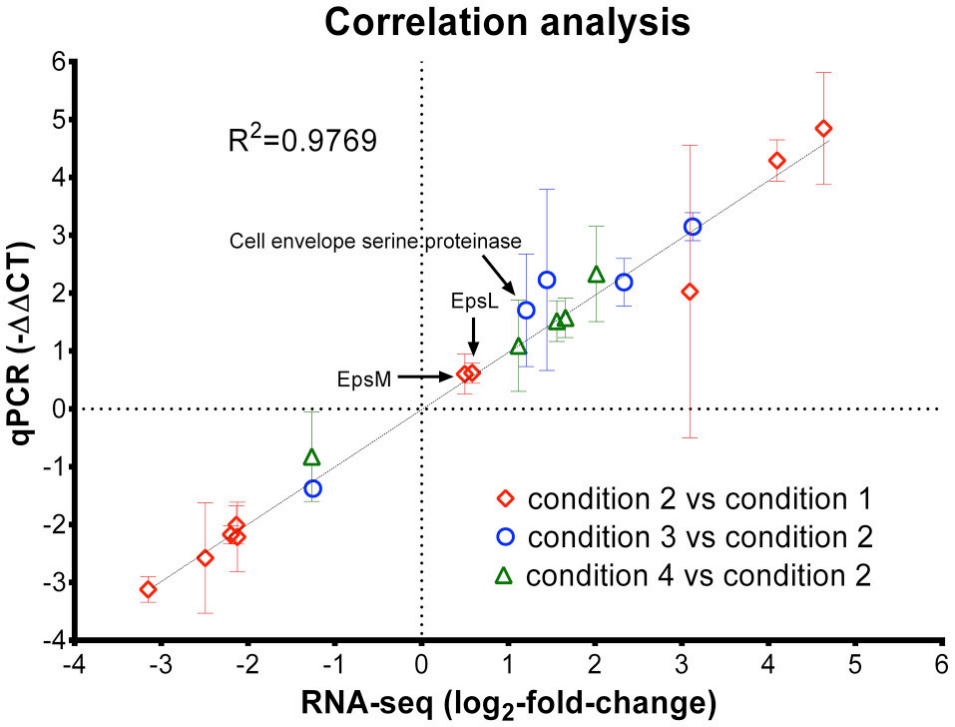
Correlation analysis of gene expression level from RNA-seq analysis and reverse-transcriptase qPCR assay. Diamond, genes selected from the comparison of condition 2 and condition 1 (pH effect); Circle, genes selected from the comparison of condition 3 and condition 2 (temperature effect); triangle, genes selected from the comparison of condition 4 and condition 2 (WPI effect). The error bar (standard deviation) for -ΔΔC_T_ (qPCR assay) was indicated; only the mean values for qPCR assay and RNA-seq results were used for linear regression analysis.

Next, reverse-transcriptase qPCR assay was used to confirm the gene expression of 20 genes with variable fold changes by RNA-seq. Twenty genes including 10 from Cd2 vs. Cd1 comparison, 5 from Cd3 vs. Cd2 comparison and 5 from Cd4 vs. Cd2 comparison were selected for qPCR verification. The reference gene (T303_03540; Table 1), *tuf*, was used for normalization of gene expression in ST1275 during qPCR assay (Li et al., 2011); the count-per-million values of this gene under the four conditions tested were very similar indicating its highly stable expression in ST1275 (Table S1). Thus, *tuf* was selected as the reference gene for normalization purposes for qPCR assay. As shown in Figure 2, the correlation coefficient (r) was about 0.9769 indicating that the fold changes of selected genes from RNA-seq and qPCR assay were strongly and positively correlated, in the other words, qPCR results confirmed the expression levels of these 20 genes via RNA-seq analysis. Since these 20 genes had variable fold changes (Figure 2), this may generally support the accuracy of RNA-seq results. However, RNA-seq over-performs qPCR assay in terms of detection limit, detection accuracy and data interpretation (FDR analysis); thus, we believe that RNA-seq method in this study could provide us accurate transcriptome results for ST1275.

### Nucleotide sugar synthesis

**Figure 3:**
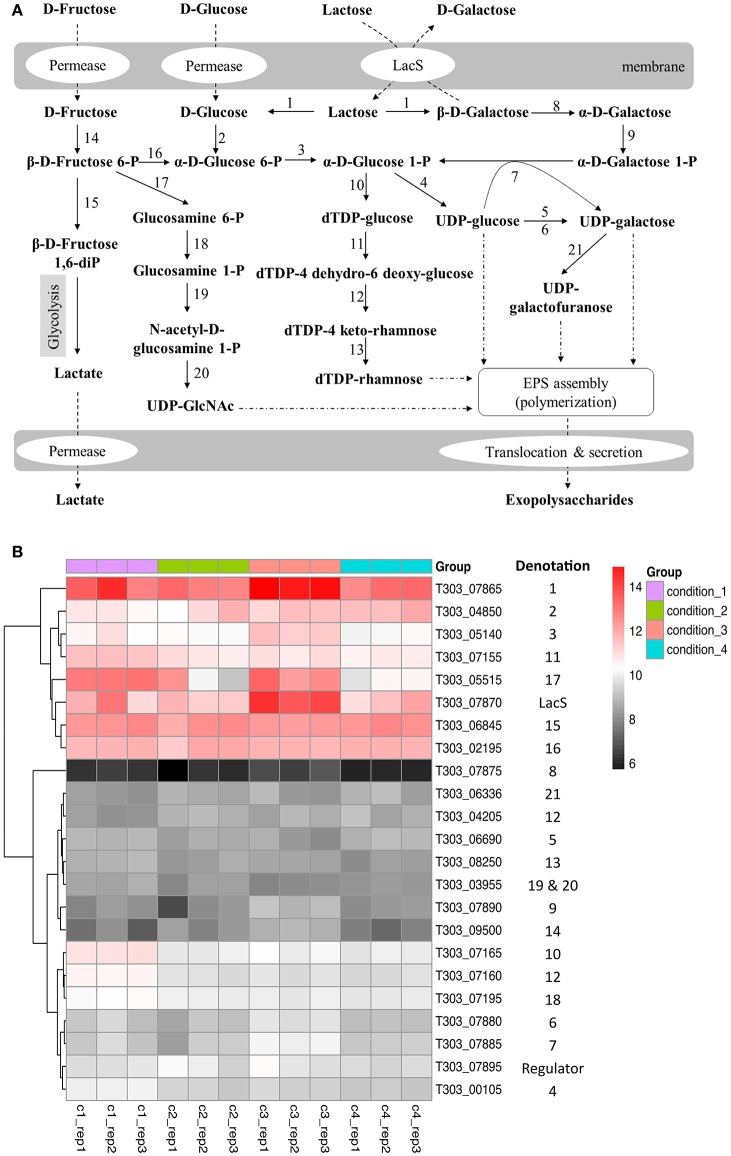
Changes in gene expression of nucleotide sugar synthesis-associated genes in ST1275 under different conditions. **(A)** biosynthesis pathways of nucleotide sugars. **(B)** Heatmap of expression level (log_2_CPM) of nucleotide sugar synthesis-associated genes. Denotations: 1, β-Galactosidase; 2, Glucokinase; 3, Phosphoglucomutase; 4, UDP-glucose pyrophosphorylase; 5, UDP-glucose 4 epimerase; 6, UDP-galactose 4 epimerase; 7, Galactose 1-phosphate uridylytransferase; 8, Galactose mutarotase; 9, Galactokinase; 10, dTDP-glucose pyrophosphorylase; 11, dTDP-glucose-4, 6-dehydratase; 12, dTDP-4 keto-6 deoxy-glucose 3, 5-epimerase; 13, dTDP-4 keto-L-rhamnose reductase; 14, Fructokinase; 15, 6-phosphofructokinase; 16, Phosphoglucose isomerase; 17, glutamine-fructose-6-phosphhate transaminase; 18, Phosphoglucosamine mutase; 19 and 20, N-acetylglucosamine-1-phosphate uridyltransferase (bifunctional); 21, UDP-galactopyranose mutase.

The effects of pH, temperature and WPI on the gene expression of nucleotide sugar synthesis are presented in Figure 3, Table S1. For Cd2 vs. Cd1 (pH effect), the pH of milk medium that decreased from pH 6.5 to pH 5.5 down-regulated expression level of genes (T303_07160, T303_07165, T303_07195) that are associated with dTDP-rhamnose and UDP-GlcNAc (Figure 3B). The expression of glycolysis-associated genes such as T303_07865, T303_04850, T303_02195 and T303_06845 did not change much (Cd2 vs. Cd1). As for temperature effect (Cd3 vs. Cd2), the temperature of milk fermentation that increased from 37 to 40°C significantly up-regulated the expression of genes associated with lactose catabolism, UDP-glucose/UDP-galactose synthesis and UDP-GlcNAc synthesis (Figure 3B, Table S1). There were no significant changes in the expression level of nucleotide sugar synthesis-associated genes in ST1275 in milk supplemented with WPI (Cd4 vs. Cd2; Table S1).

### EPS assembly

**Figure 4:**
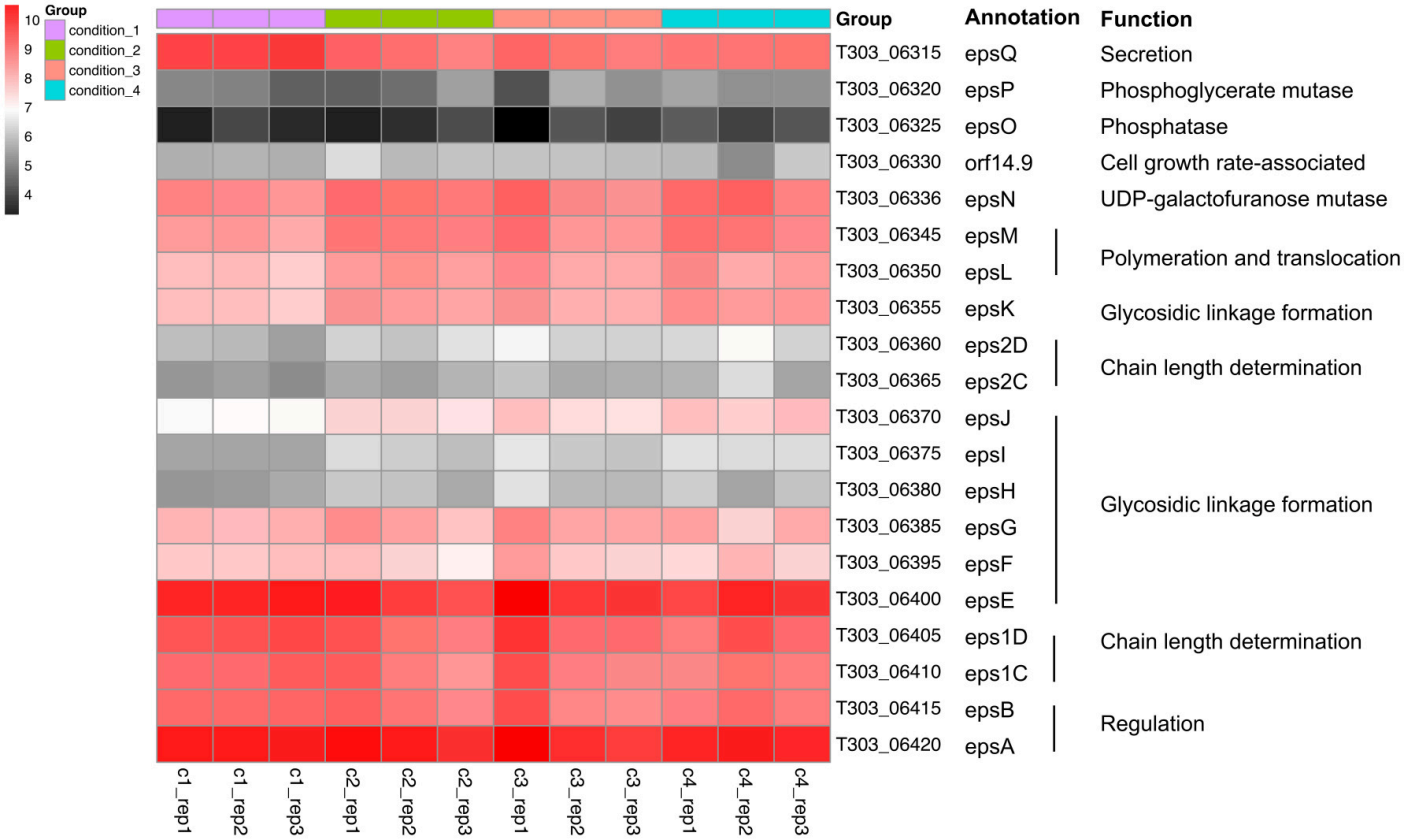
Heatmap of expression level (log_2_CPM) of EPS assembly-associated genes in ST1275 under different conditions.

The changes in gene expression of *eps* gene cluster are shown in Figure 4, Table S1. Significant DEGs were only observed when assessing the effect of pH drop (Cd2 vs. Cd1; Table S1): (1) *epsM* and *epsL* related with EPS polymerization and membrane translocation, and has been validated by qPCR; (2) *epsK, epsI*, and *epsJ* related with glycosidic bond formation by glycosyltransferase. The gene expression of *epsQ*, assigned for the transfer of EPS between the membrane and peptidoglycan layer (Wu et al., 2014), were significantly down-regulated suggesting the EPS exportation may be reduced. However, as the milk fermentation is a long-term process that gives enough time for cells to release EPS. As presented in Figure 4, a similar expression pattern (from T303_06320 to T303_06385) was observed in Cd2, Cd3, and Cd4 compared to that in Cd1; this suggests that temperature increase (Cd3 vs. Cd2) and WPI supplementation (Cd4 vs. Cd2) did not alter the expression of these genes, whereas pH drop (Cd2 vs. Cd1) up-regulated the genes associated with EPS assembly and translocation.

So far, the two *epsC-epsD* gene sets were only observed in ST1275 among the sequenced strains of *Str. thermophilus* (Wu et al., 2014). Interestingly, two unique *eps1C-eps1D* and *eps2C-eps2D* assigned for determining the chain length of EPS repeating units showed opposite expression pattern under two different pH conditions (Figure 4). For additional temperature increase, the *eps1C-eps1D* gene expression was also increased under Cd3 compared to that under Cd2. This indicates that the polymerization degree of EPS may vary from condition to condition.

### Arginine, methionine, and cysteine metabolism

**Figure 5:**
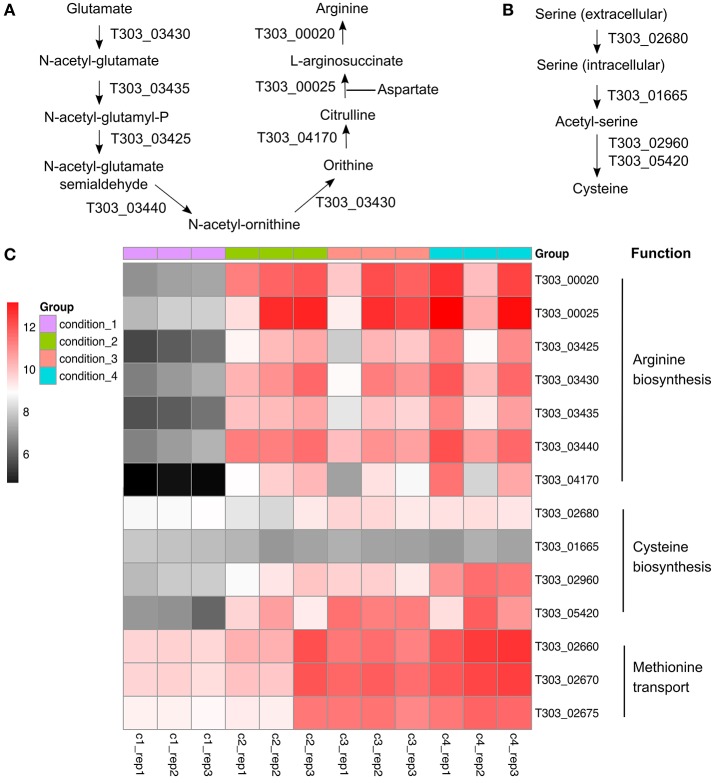
Gene expression profile (log_2_CPM) of arginine/cysteine/methionine-associated genes in ST1275 under different conditions. **(A)** arginine synthesis pathway; **(B)** cysteine synthesis pathway; **(C)** Heatmap of expression level (log_2_CPM) of arginine, cysteine, and methionine-associated genes.

The changes in gene expression of arginine/methionine/cysteine metabolism-associated genes are presented in Figure 5, Table S1. Acidic condition (pH 5.5) significantly altered the gene expression of arginine/methionine/cysteine metabolism-associated genes, especially arginine biosynthesis. WPI supplemented to milk also increased the level of mRNA transcripts of arginine/methionine/cysteine metabolism-associated genes in ST1275.

### Proteolytic activity

As shown in Table S1 (log2CPM spreadsheet), the only one gene (T303_05205) encoding extracellular serine proteinase—lactocepin was up-regulated in Cd3 and Cd4 conditions as compared to Cd1 and Cd2. This may indicate an improved extracellular proteolytic activity of ST1275 under Cd3 and Cd4 in milk environment.

## Discussion

EPS production machinery in ST1275, a typical conventional dairy starter, could be stimulated under optimal conditions, i.e., pH 5.5, 37 or 40°C and WPI supplementation, for EPS biosynthesis and release. Thus, genome-wide comparative transcriptome strategy was applied to obtain global changes in cellular transcription in this organism under different pH and temperature when cultured in milk environment supplemented with or without WPI. The high-quality RNA-seq data provided sufficient depth and coverage for calculating the abundance of detected coding genes (Tables 2, 3). In addition, qPCR assay also verified the accuracy of RNA-seq data (Figure 2). It was noted that several monosaccharides including mannose, galactose, glucose, fucose, glucosamine and galactosamine in the EPS of ST1275 were detected by HPLC equipped with a UV detector based on the retention time of standards (Li and Shah, 2016). This may be not accurate due to two reasons: (1) this detection is not based on mass spectroscopy; (2) there are no pathways for synthesis of nucleotide sugars such as UDP-GlcA, UDP-GalA, GDP-mannose and GDP-fucose based on our genetic annotations (Wu et al., 2014) and this transcriptome study–functional approach. However, it is advised that glucose, galactose, rhamnose and GlcNAc are commonly detected in the EPS from *Str. thermophilus* (Lemoine et al., 1997; Degeest et al., 2002; Broadbent et al., 2003; Vaningelgem et al., 2004; Tabibloghmany and Ehsandoost, 2014).

### Effects of pH decrease (Cd2 vs. Cd1) on global cellular transcription

As more EPS was produced from ST1275 under pH 5.5 compared to that under pH 6.5, the pH effect (Cd2 vs. Cd1) on genome-wide cellular transcription was first assessed. Although there was 600 DEGs accounted for about one third of coding genes in ST1275 (Figure 1D, Table 4), it is clear that the pH decrease resulted in the dramatic shift of gene expression profiles accounted for enhanced EPS production (Figure 1B,C). Among them, EPS assembly/translocation were up-regulated under pH 5.5 (Figure 4). It has been indicated that almost 3-fold increase in the gene expression of *eps* gene cluster resulted in 4-fold EPS production from *L. lactis* (Boels et al., 2003). Thus, the increased mRNA level of *eps* gene cluster (*epsNMLKJ*) may support the enhanced EPS production from ST1275 in such acidic condition. However, dTDP-rhamnose and UDP-GlcNAc synthesis-associated genes were significantly down-regulated (Figure 3, Table S1); this alteration in EPS precursors may result in the changes in chemical composition of EPS, though the EPS production from ST1275 increased under pH 5.5. Thus, this merits further chemical characterization of EPS from different conditions.

### Effects of temperature increase (Cd3 vs. Cd2) on global cellular transcription

Lactose is the main carbohydrate in milk and *Str. thermophilus* transports lactose via the LacS (lactose permease) that also function as a lactose/galactose antiporter or a galactose/proton symporter (Foucaud and Poolman, 1992). Temperature increase from 37 to 40°C (Cd3 vs. Cd2) had limited effects on the cellular transcription as evidenced by only 23 DEGs (Figure 1E, Table S1). Among them, the improved gene expression of lactose catabolism-associated genes may result in more nucleotide sugars for EPS assembly (Figure 3). This has been demonstrated in *Str. thermophilus* strain LY03 by (Levander et al., 2002). In addition, EPS-assembly-associated genes were also enhanced (Figure 4), thus this may support better EPS production from ST1275 cultivated in a higher temperature (Boels et al., 2003). Importantly, the genes encoding cell envelope-associated proteinase were up-regulated in Cd3 compared to Cd2; this may indicate a better proteolytic activity in the condition of 40°C as extracellular proteinase of *Str. thermophilus* plays a critical role in adapting to milk environment (Hols et al., 2005; Delorme et al., 2010; Hafeez et al., 2013). It appears that the condition 3 (Cd 3; pH 5.5 and 40°C) showed the highest potential for enhanced EPS production based on those important gene expression patterns. Although limited alterations in dairy fermentation for certain type of products are allowed, simple changes in pH and temperature parameters may be allowed for new fermented dairy products.

### Effects of WPI supplementation (Cd4 vs. Cd2) on global cellular transcription

Only 9 DEGs (Figure 1F) were obtained when assessing the WPI effect. Most of these DEGs are associated with EPS assembly and arginine, methionine, and cysteine synthesis (Figure 4,5). As mentioned above, enhanced mRNA level of *eps* gene cluster may support higher EPS production (Boels et al., 2003). Evidence indicates minimal sulfur amino acid auxotrophy (histidine/methionine/cysteine) of *Str. thermophilus* LMG18311 in chemically defined medium suggesting the roles of methionine/cysteine involved in the optimal growth of *S. thermophilus* (Pastink et al., 2009). Meanwhile, it has been reported that methionine is a rare amino acid in milk (Tamine and Deeth, 1980). Thus, an increase in cysteine synthesis and methionine transport from milk environment (WPI) under condition 4 may help ST1275 cells grow in milk. Furthermore, sulfur volatile compounds synthesized from methionine/cysteine in dairy starters provide characteristic flavor to fermented milk products (Liu et al., 2012). It appears that fermented milk from condition 4 may have a better flavor. Although the arginine synthesis-associated genes were up-regulated significantly (Figure 5), the function of arginine in ST1275 is still not well documented. There was one study describing the role of arginine in cytosolic pH homeostasis via the arginine decarboxylase-urease pathway (Huang et al., 2016). However, this pathway is absent in ST1275 (refer to KEGG website for this strain). In addition, whey proteins are rich in glutamate and aspartate which are substrates for arginine biosynthesis (Figure 5A); this may suggest an enhanced arginine production due to the supplementation of WPI. Further investigations on arginine function in ST1275 and its role in EPS production are necessary.

### The regulation of two sets of *epsC*-*epsD* in ST1275

The *epsC*-*epsD* was assigned for chain length determination and export (Goh et al., 2011). In general, EpsC is required for tyrosine phosphorylation of EpsD resulting in a EpsD phosphorylated form, whereas the activity of EpsE, the priming enzyme for EPS repeating unit synthesis, is activated by the phosphorylated EpsD (Minic et al., 2007). We previously identified two sets of *epsC*-*epsD* in ST1275 which is rare in *Str. thermophilus* (Wu et al., 2014). Now, it is the first time that we confirmed the transcription levels of both *epsC*-*epsD* sets in this organism (Figure 4). Notably, the transcription level of *eps1C*-*eps1D* and *epsE* did not change much among four conditions, whereas *eps2C*-*eps2D* was slightly up-regulated under Cd2, Cd3, and Cd4 compared to Cd1; this may suggest a secondary regulation for EpsE occurred in Cd2, Cd3, and Cd4 (Figure 4) resulting in different chain lengths of EPSs produced from ST1275. Moreover, EpsB exhibits phosphatase activity against phosphorylated EpsD (Bender and Yother, 2001; Minic et al., 2007); thus EpsB acts as a modulator of EpsD as evidenced by the enhanced EpsE activity in the mutant *Str. thermophilus* strain lacking EpsB (Minic et al., 2007). Hence, the slightly down regulation of *epsB* (inhibition) and up-regulation of *eps2D* (activation) may result in an improved EpsE activity that supports the improved EPS yields from ST1275 under Cd2, Cd3, and Cd4 compared to that under Cd1.

## Conclusions

The genome-wide transcriptome analysis for high EPS-producing ST1275 under various fermentation conditions provided detailed global insights into the gene expression patterns that were associated with enhanced EPS yields. Particularly, up-regulation of genes that were related with nucleotide sugars synthesis, EPS assembly, arginine, methionine, and cysteine synthesis or transport and proteolysis would be accounted for improving EPS production from ST1275. This study also indicates that EPS production from *Str. thermophilus* involves many synthesizing genes (i.e., *eps* gene cluster) that could be modulated by environmental conditions such as pH to cooperate together for an enhanced EPS yield.

## Author contributions

QW and NS designed the study. QW performed the experiments excluding the mRNA sequencing service, analyzed the data, and generated the figures and tables. QW and NS wrote the paper.

### Conflict of interest statement

The authors declare that the research was conducted in the absence of any commercial or financial relationships that could be construed as a potential conflict of interest.

## Supplementary material

Figure S1 Re-analysis of data generated from our 2003 pioneer study (Zisu and Shah, 2003). **(A)** principal component analysis (PCA) of EPS data from ST1275 during 24-h time-course milk fermentation; **(B)** PCA analysis of bacterial cell viability of ST1275 during 24-h time-course milk fermentation. WPC, whey protein concentrate; PCA, principal component analysis.

Table S1 List of genes with their expression level in ST1275 under the four conditions assessing the effects of pH, temperature and WPI.
