# Supplementary figures and images for: Comparative mRNA-Seq Analysis Reveals the Improved EPS Production Machinery in Streptococcus thermophilus ASCC 1275 During Optimized Milk Fermentation
Source: Front Microbiol. 2018 Mar 13;9:445. doi: 10.3389/fmicb.2018.00445 (PMC5859087; doi:10.3389/fmicb.2018.00445)

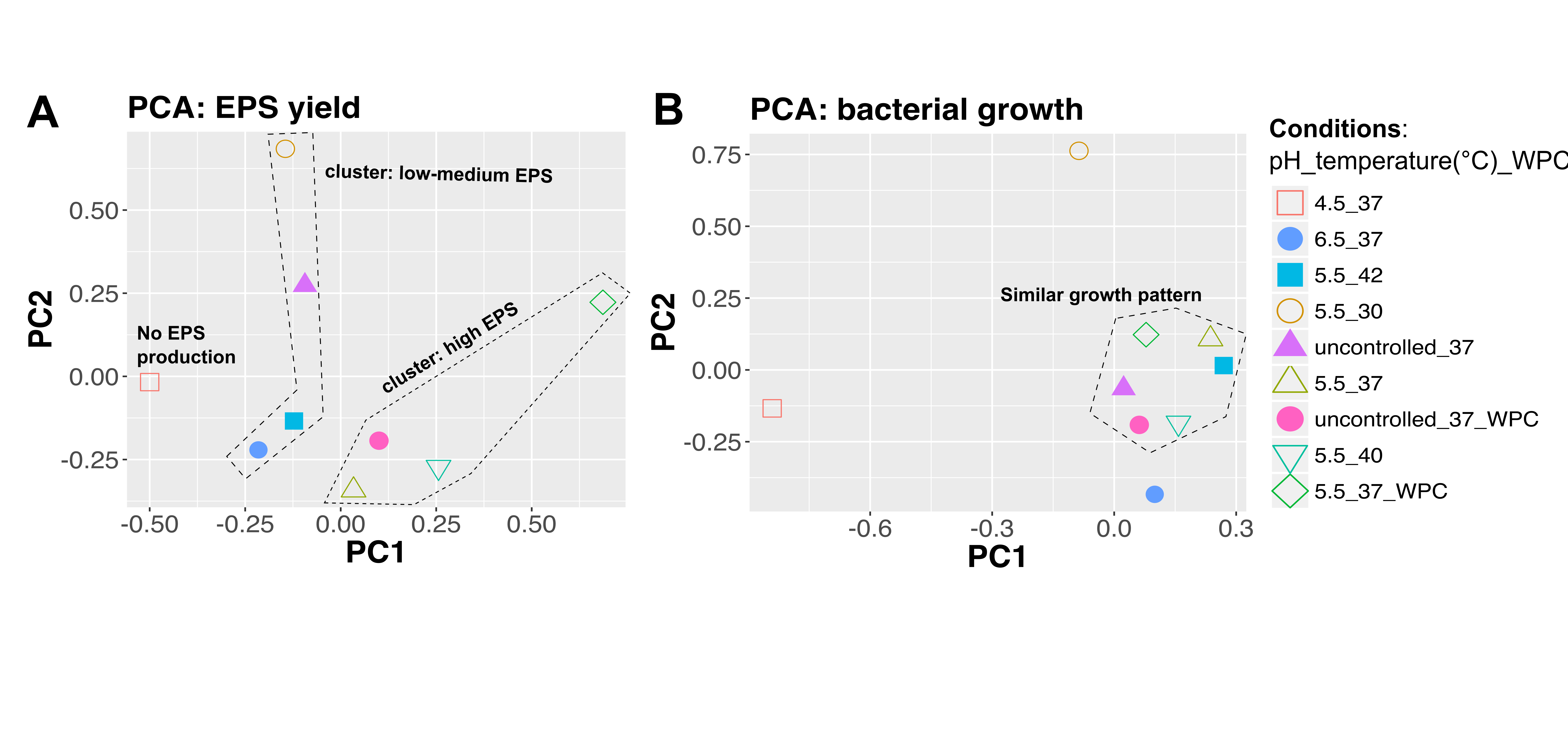

Supplement: Figure S1 — Re-analysis of data generated from our 2003 pioneer study (Zisu and Shah, 2003). (A) principal component analysis (PCA) of EPS data from ST1275 during 24-h time-course milk fermentation; (B) PCA analysis of bacterial cell viability of ST1275 during 24-h time-course milk fermentation. WPC, whey protein concentrate; PCA, principal component analysis. [file Image1.JPEG]
